# Supplementary figures and images for: Voxelwise Encoding Models Show That Cerebellar Language Representations Are Highly Conceptual
Source: J Neurosci. 2021 Dec 15;41(50):10341–55. doi: 10.1523/JNEUROSCI.0118-21.2021 (PMC8672691; doi:10.1523/JNEUROSCI.0118-21.2021)

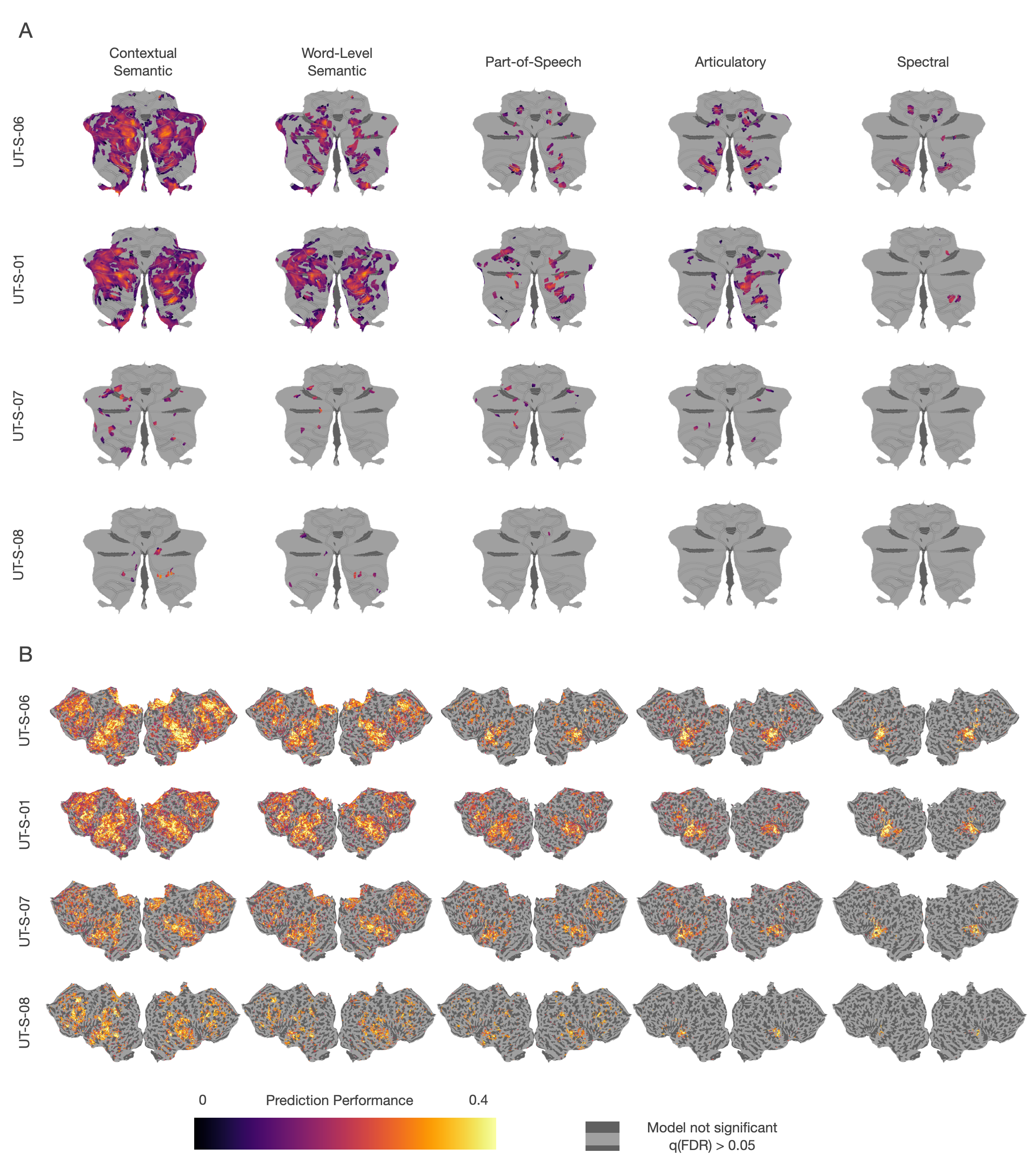

Supplement: Figure 2-1 — Prediction performance of encoding models based on five language feature spaces in cortex and cerebellum. Encoding models fit with 5.4 hours of BOLD data were tested against a held-out story (10 minutes). Correlation (r2) between predicted and actual BOLD response is plotted on flattened (A) cerebellar and (B) cortical surfaces for each. Significance testing for each model in each voxel was done using a one-sided FDR-corrected permutation test with a threshold of p<0.05. The higher-level models have better prediction performance in both cerebellum and cortex. In cortex, the areas best predicted by each of the three feature categories are spatially distinct. However, in the cerebellum, the areas best predicted by each feature space are highly overlapping. Download Figure 2-1, TIF file. [file ns-JN-RM-0118-21-s01.tif]

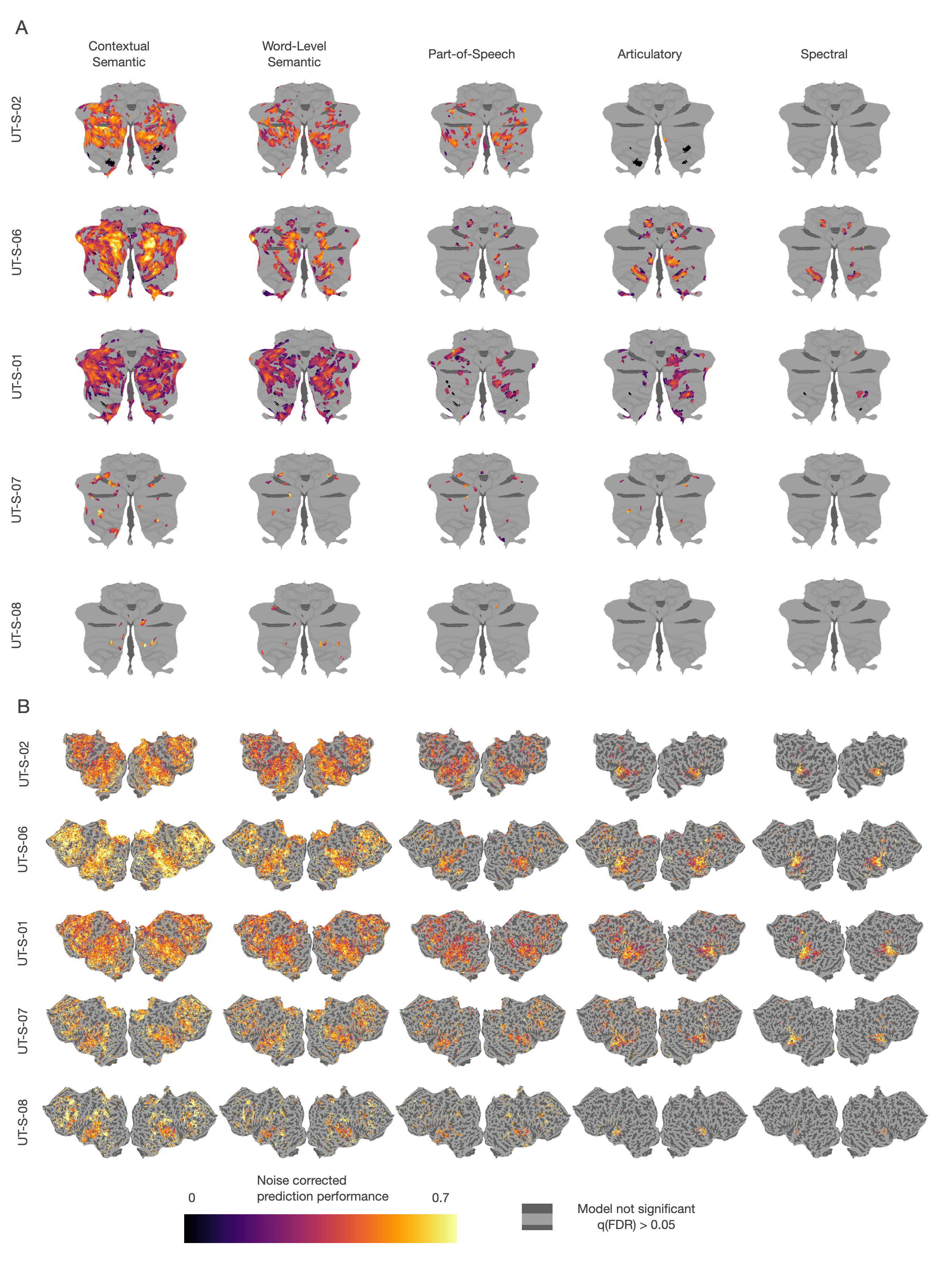

Supplement: Figure 2-2 — Prediction performance of encoding models based on five language feature spaces correcting for difference in signal-to-noise in cerebellum and cortex. Encoding models fit with 5.4 hours of BOLD data were tested against a held-out story (10 minutes). The correlation (signed r2) between predicted and actual BOLD response is plotted on flattened cerebellar (A) and cortical (B) surfaces for one subject (UT-S-02).The correlations were noise-ceiling corrected using standard techniques to account for differences in BOLD signal-to-noise ratio in the cerebellum and cortex. Significance testing for each model was done using a one-sided FDR-corrected permutation test with a threshold of p<0.05. The higher level models have better prediction performance in both cerebellum and cortex. In cortex, the areas best predicted by each of the three categories of feature spaces are spatially distinct. However, in the cerebellum the areas best predicted by each of the feature spaces is highly overlapping. This suggests that there is a hierarchy of language processing in the cortex and not in the cerebellum Download Figure 2-2, TIF file. [file ns-JN-RM-0118-21-s02.tif]

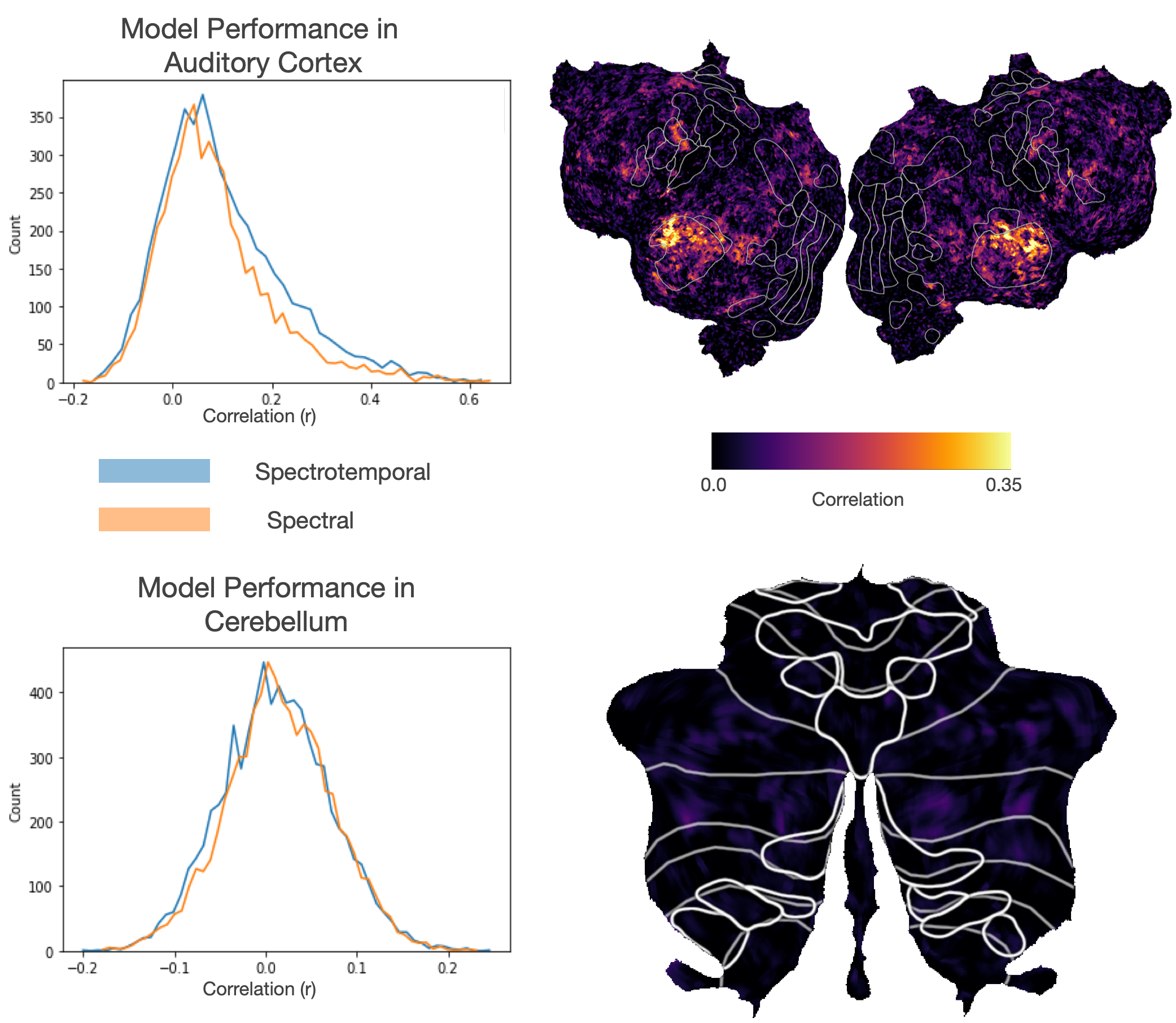

Supplement: Figure 2-3 — Spectrotemporal features do not explain more variance in cerebellum than spectrogram features. To confirm that our result is not merely due to the spectral feature space missing representations that could capture low level auditory processing in the cerebellum, we also fit the spectrotemporal model as described in Norman-Haignere et al., 2018. Here we show histograms of model prediction performance for the spectral model used elsewhere in the paper and the spectrotemporal model for cortex (top) and cerebellum (bottom) as well as their corresponding flatmaps. These results show that there is only a small benefit in using the more advanced model, mostly in the auditory cortex. The histograms show the range of model performance across voxels in the auditory cortex and cerebellum respectively. However, this spectrotemporal still fails to demonstrate any significant model prediction in the cerebellum. Download Figure 2-3, TIF file. [file ns-JN-RM-0118-21-s03.tif]

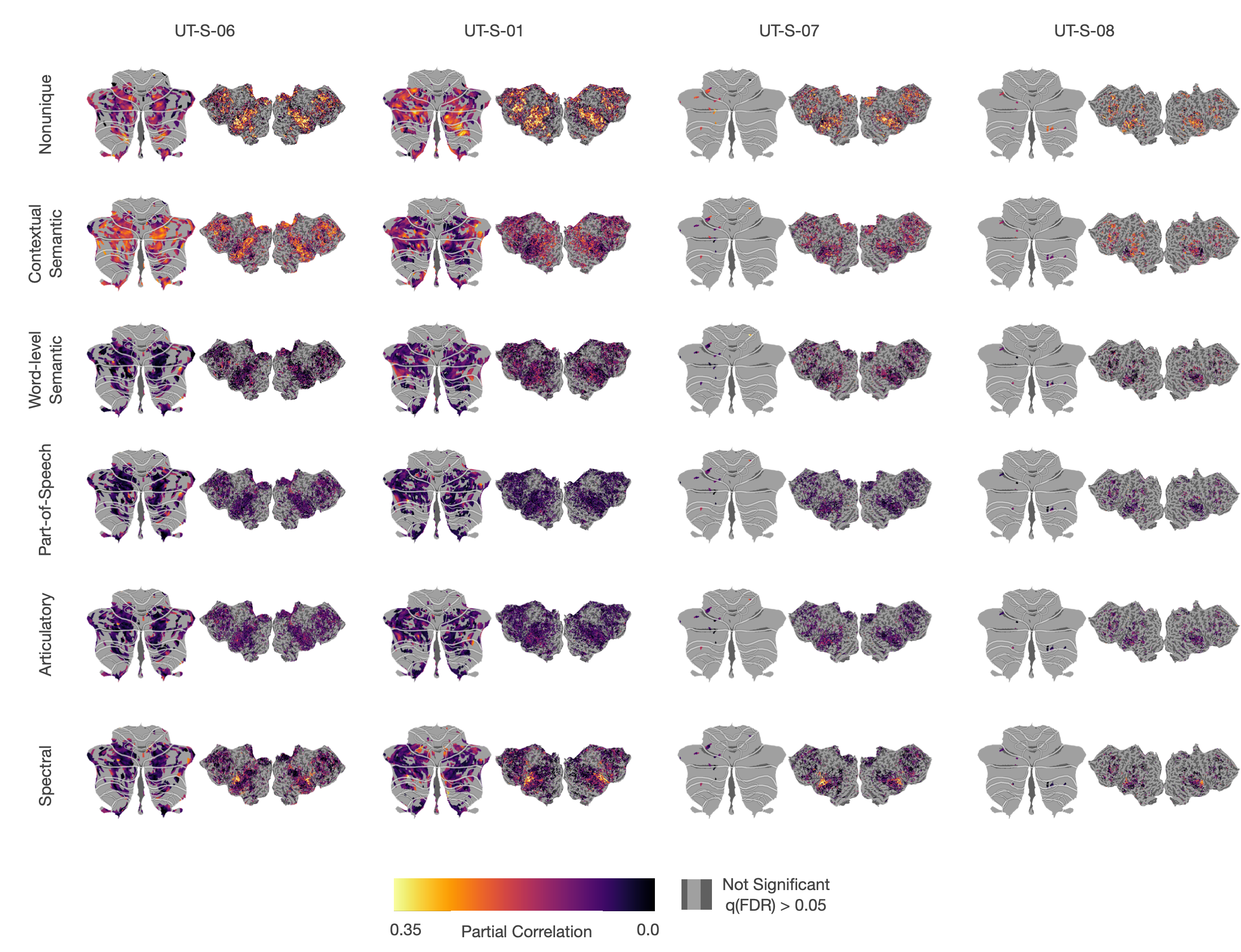

Supplement: Figure 3-1 — Unique variance explained by each feature space. To determine how much variance is uniquely explained by each feature space, six new encoding models were fit: a union model containing a concatenation of all feature spaces, and five encoding models each containing a concatenation of four of the five feature spaces. The unique contribution of each feature space was then determined by subtracting the variance explained by the four-way concatenation model without that feature space from the union model. This shows how much variance can be explained by each feature space above and beyond the other four. Additionally, the amount of nonunique variance—i.e., any that can be explained by more than one feature space—was determined by subtracting the five unique variances from the union. The voxelwise partial correlation ((partialr2)) for each feature space for each subject projected onto the cortical and cerebellar surfaces Only voxels that were significantly predicted (one-sided permutation test, q(FDR) < 0.05) by the five-way union model are displayed. Download Figure 3-1, TIF file. [file ns-JN-RM-0118-21-s04.tif]

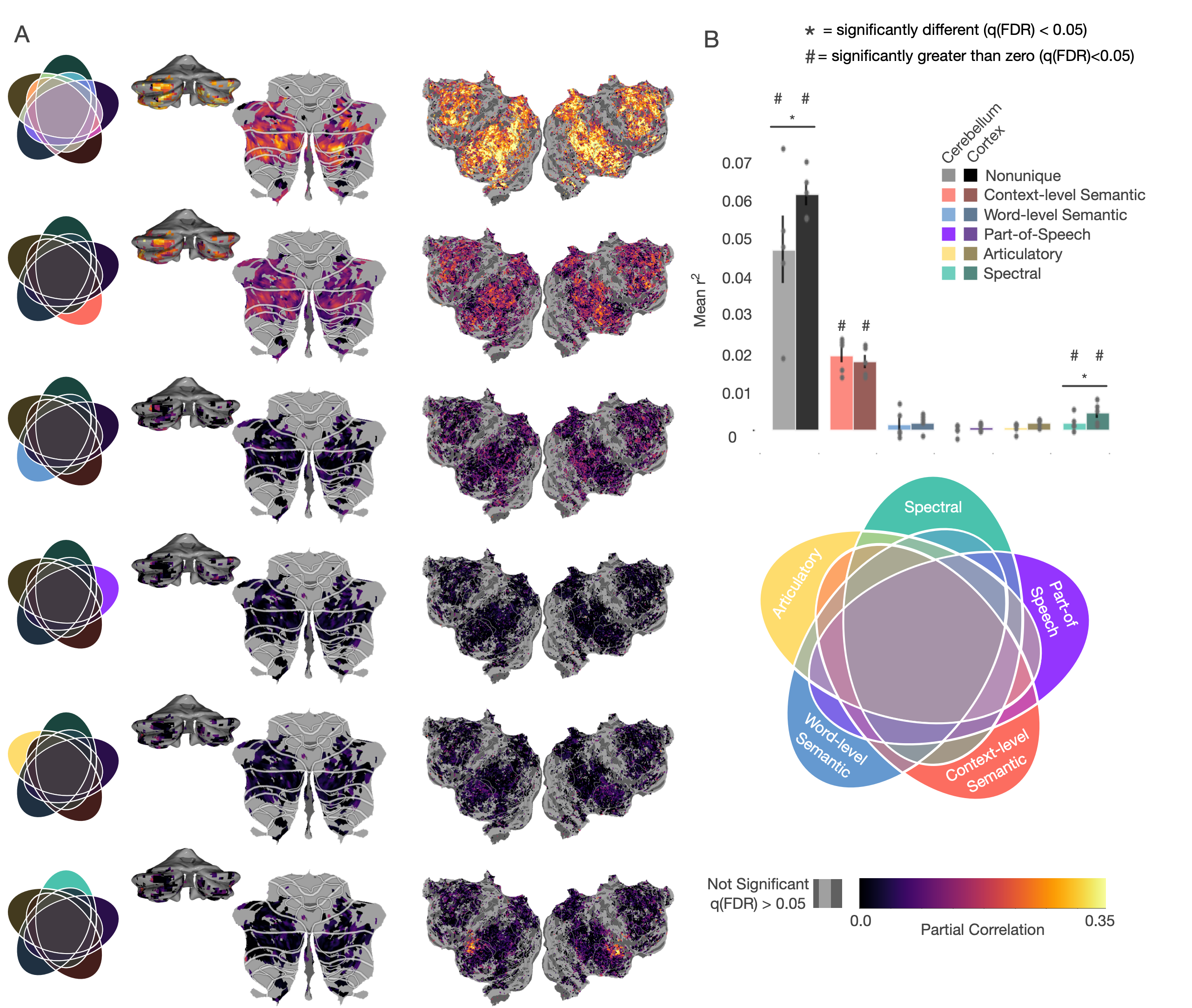

Supplement: Figure 3-2 — Unique variance explained by each feature space, not bias corrected. To determine how much variance is uniquely explained by each feature space, six new encoding models were fit: a union model containing a concatenation of all feature spaces, and five encoding models each containing a concatenation of four of the five feature spaces. This was not corrected to account for overfitting in the larger models. The unique contribution of each feature space was then determined by subtracting the variance explained by the four-way concatenation model without that feature space from the union model. This shows how much variance can be explained by each feature space above and beyond the other four. Additionally, the amount of nonunique variance—i.e., any that can be explained by more than one feature space—was determined by subtracting the five unique variances from the union. (A) The voxelwise partial correlation ( r2) for each feature space for Subject UT-S-02, projected onto the cortical and cerebellar surfaces. Only voxels that were significantly predicted (one-sided permutation test, q(FDR) < 0.05) by the five-way union model are displayed. (B) Mean correlations for significant voxels in the cerebellum and cortex across all subjects. The nonunique partition contains the most variance in both cortex (darker) and cerebellum (lighter). All models, except the contextual semantic model, explain less variance in cerebellum than cortex (two-sided permutation test, q(FDR) < 0.05). Only the context-level semantic model and the spectral model explain significantly >0 variance in the cerebellum. Additionally, the modality-specific feature spaces do not uniquely explain any significant variance (two-sided permutation test, q(FDR) < 0.05), while the context-level semantic space uniquely explains the most variance. This further supports the hypothesis that the cerebellum is largely representing language at a high, conceptual level. Download Figure 3-2, TIF file. [file ns-JN-RM-0118-21-s05.tif]

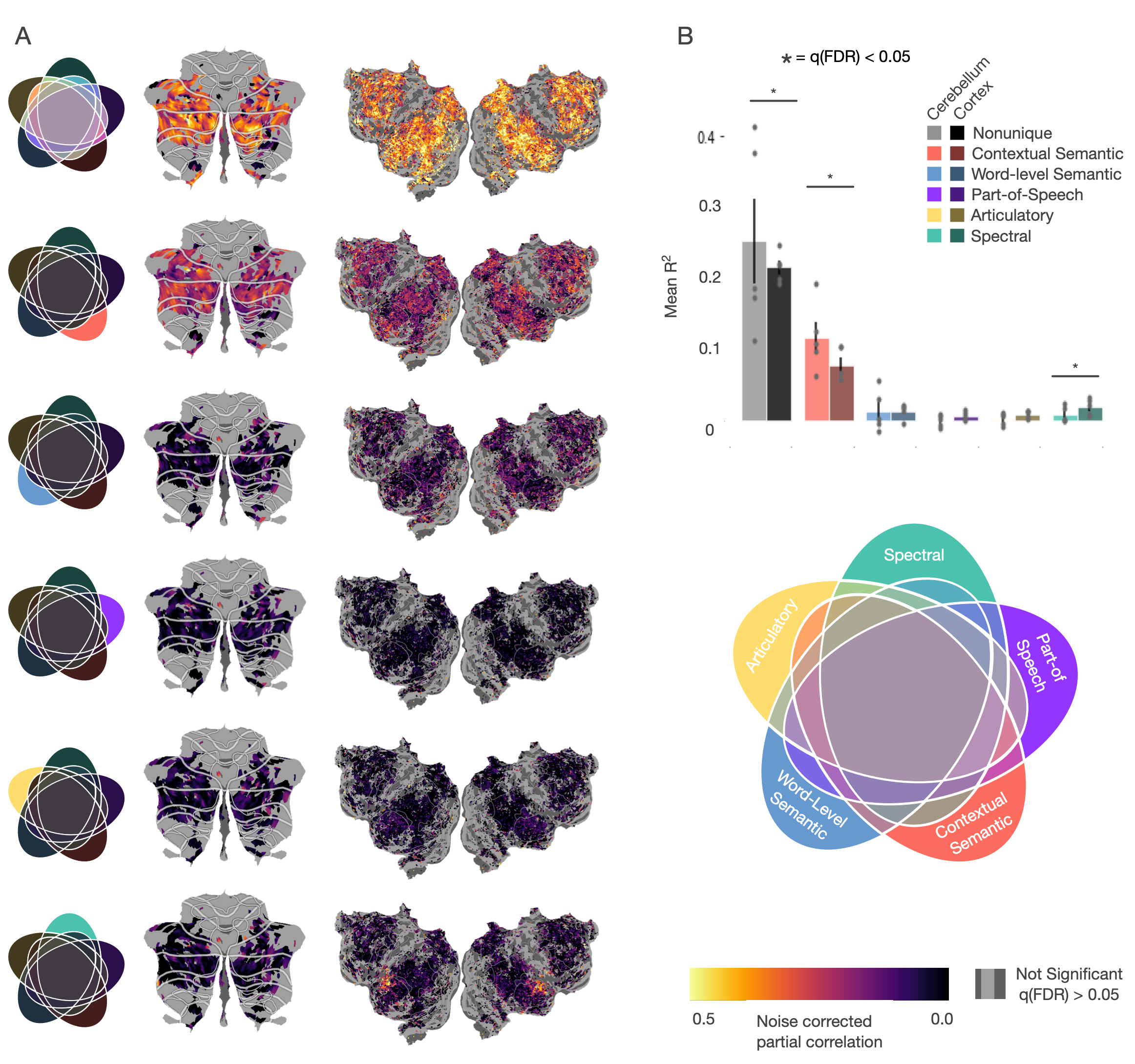

Supplement: Figure 3-3 — Unique variance explained by each feature space adjusted for differences in signal-to-noise in cerebellum and cortex. To determine the unique variance explained by each feature space, six new encoding models were fit: a union encoding model containing a concatenation of all feature spaces, and five encoding models each containing a concatenation of four of the five feature spaces. The unique contribution of each feature space was then determined by subtracting the variance explained by the four-way concatenation model without that feature space from the union model. This shows how much variance can be explained by each feature space above and beyond the other four. Additionally, the amount of nonunique variance—i.e., that which can be explained by more than one feature space—was determined by subtracting the five unique variances from the union. To account for differences in signal-to-noise between cerebellum and cortex, the correlations were corrected using standard noise-ceiling correction techniques. (A) The voxelwise partial correlation ( r2) for each feature space for Subject UT-S-02 was projected onto the cortical and cerebellar surfaces. Only voxels that were significantly predicted (one-sided permutation test, q(FDR) < 0.05) by the five-way union model are displayed. (B) Mean correlations for significant voxels in the cerebellum and cortex across all subjects. The nonunique partition explains the most variance in both cortex (darker) and cerebellum (lighter), although the variance explained by the nonunique partition is significantly (two-sided permutation test, q(FDR) < 0.05) larger in cerebellum. The modality-specific spectral feature space explains significantly less variance in the cerebellum as compared to the cortex. Additionally, the modality-specific and language-specific feature spaces explain a negligible amount of variance in the cerebellum and the context-level semantic space explains the most variance among the unique partitions in cerebellum. T [file ns-JN-RM-0118-21-s06.tif]

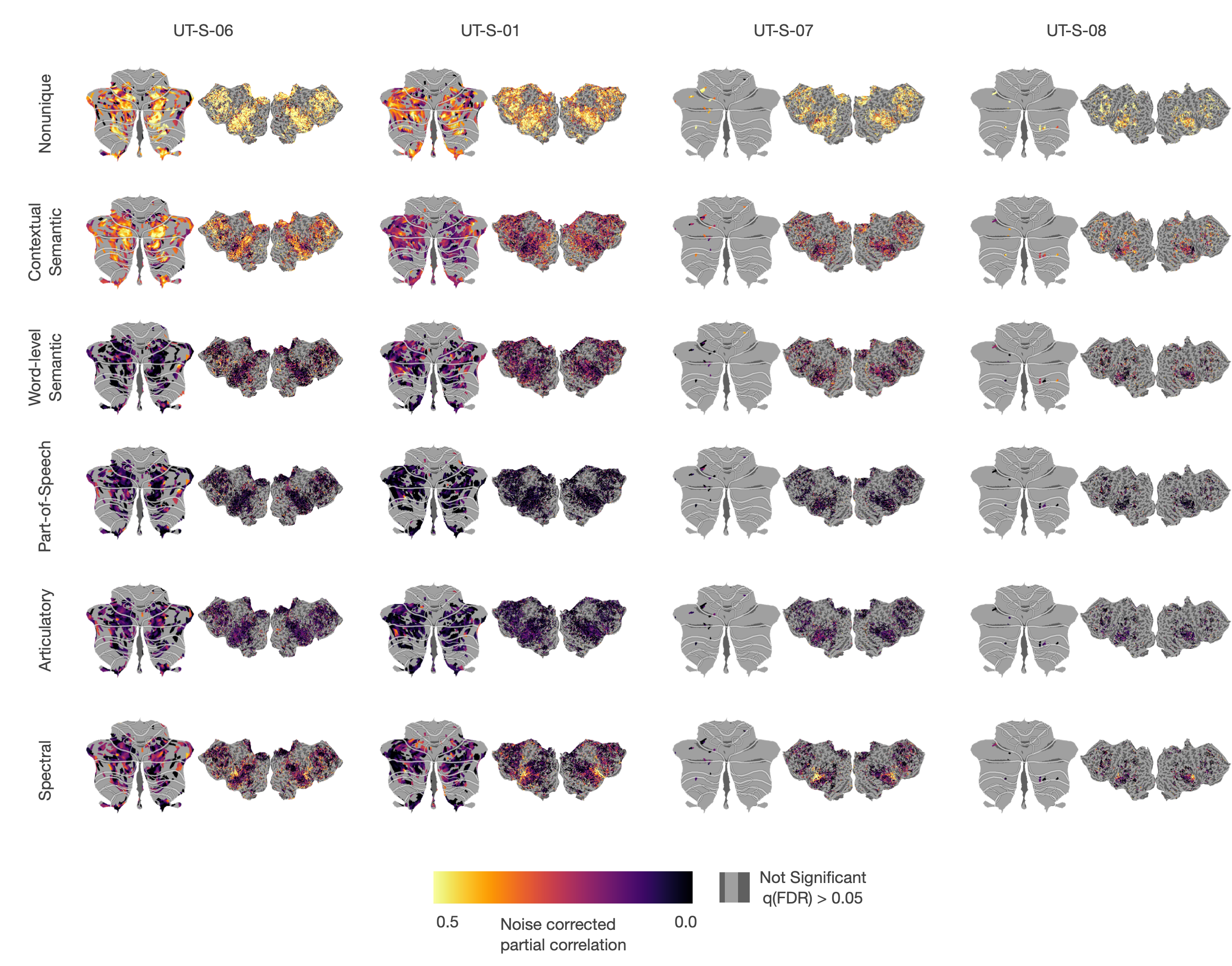

Supplement: Figure 3-4 — Unique variance explained for each feature space for additional subjects adjusted for differences in signal-to-noise in cerebellum and cortex. To determine the unique variance explained by each feature space, a union encoding model was fit with a concatenation of all feature spaces in addition to five other encoding models - each a concatenation of four of the feature spaces. The unique contribution of a model can be determined by the subtraction of the four way concatenation model without that feature space from the five way union model. Additionally, the amount of overlap between the feature spaces can be characterized by the non unique partition. The unique variance explained by each feature space in each additional subject were projected onto the cortical and cerebellar surfaces. Only significant (one-sided permutation test, q(FDR) < 0.05) voxels from the five-way joint model are displayed. Download Figure 3-4, TIF file. [file ns-JN-RM-0118-21-s07.tif]

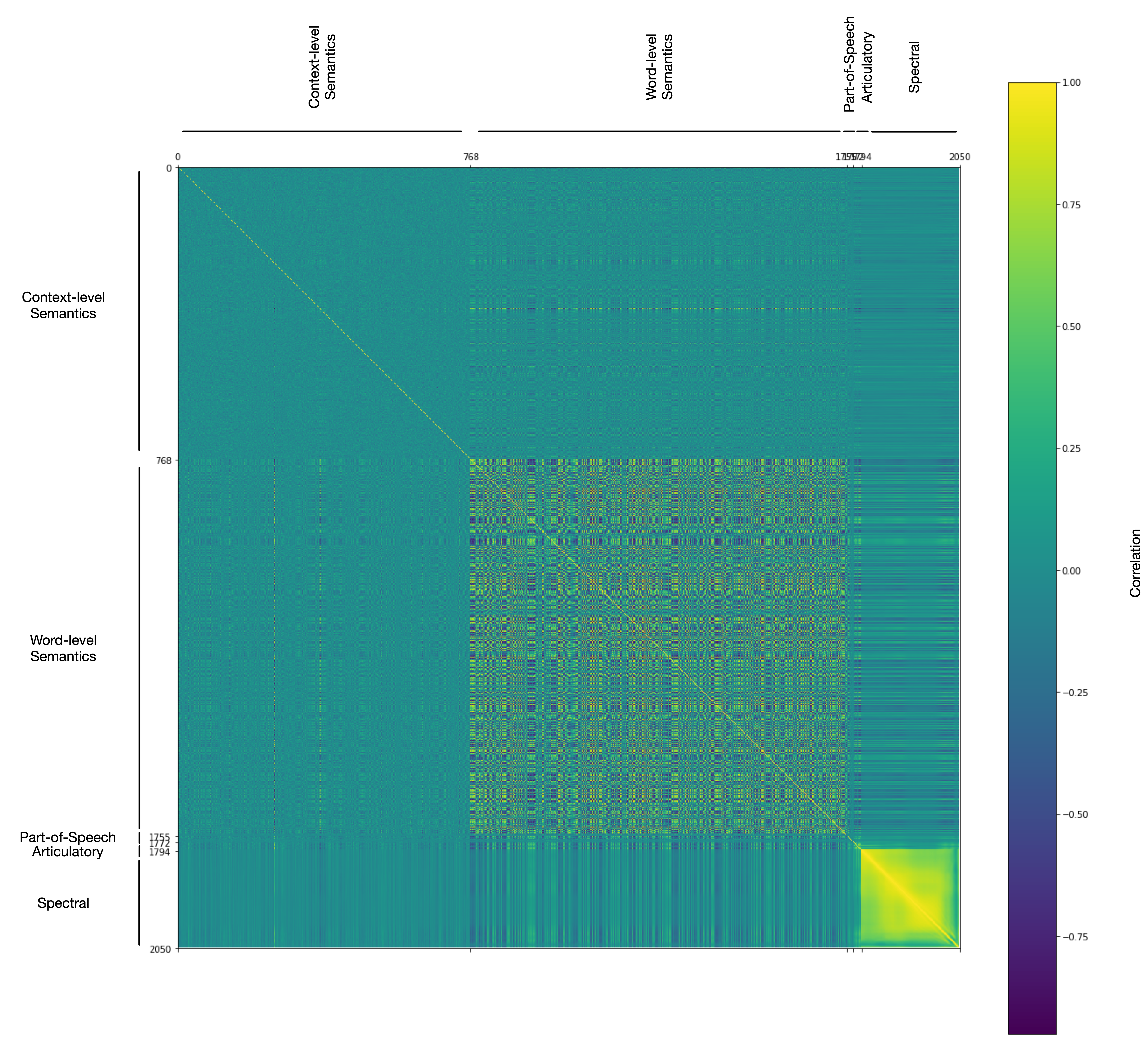

Supplement: Figure 3-5 — Feature Spaces Correlation. The feature spaces used in the encoding models often have features within them or across spaces that are correlated with each other. To demonstrate how correlated these feature spaces are, the correlations between each pair of features from all feature spaces was calculated. The context-level semantic features are relatively uncorrelated with each other and with the other features. In contrast, the word-level semantic features are more correlated with each other, and with the other feature spaces. The spectral features are very highly correlated with each other. The apparent correlations between feature spaces are likely caused by many factors. For example, periods of speech versus periods of silence should be discernible from every one of the feature spaces. Download Figure 3-5, TIF file. [file ns-JN-RM-0118-21-s08.tif]

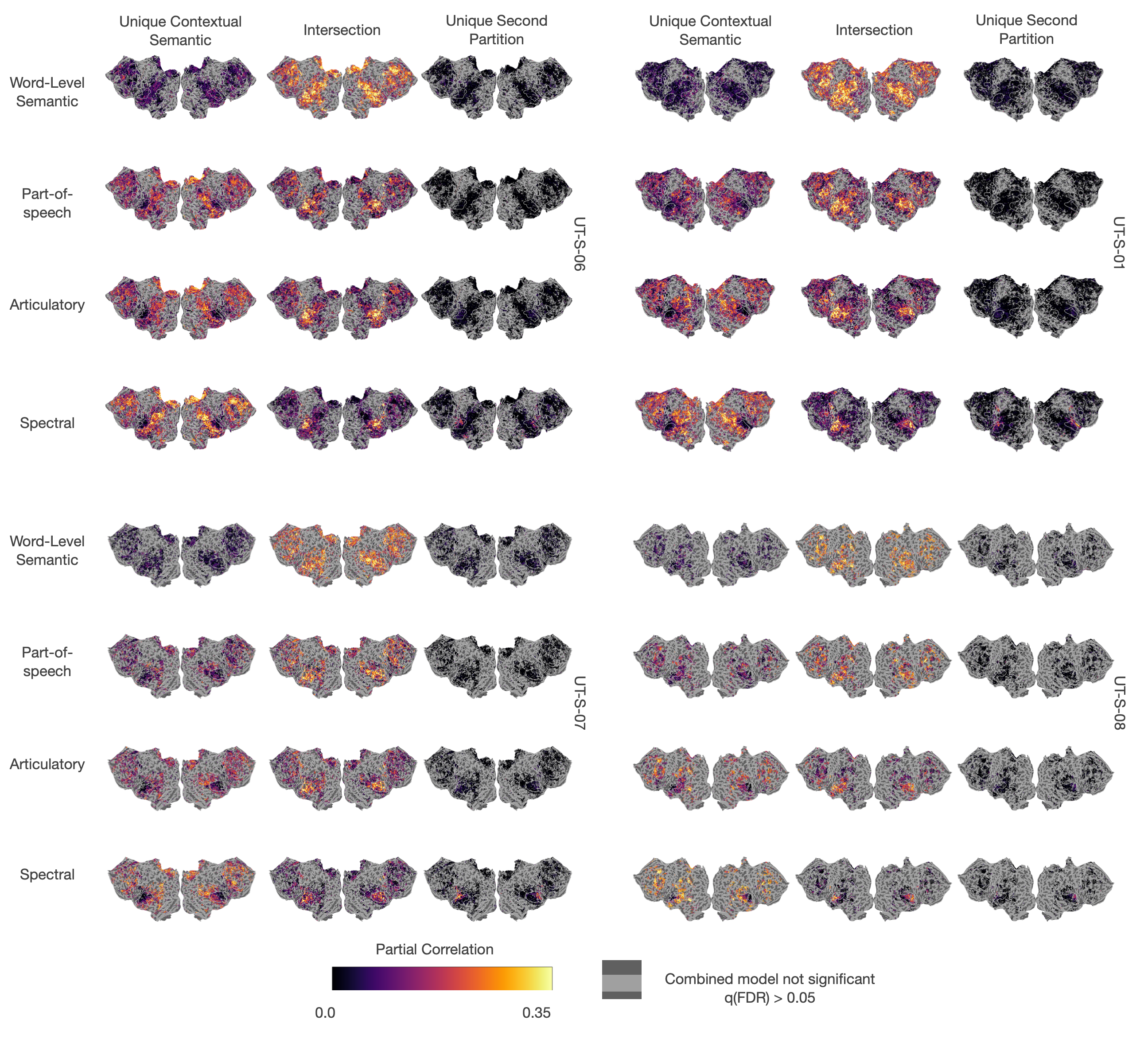

Supplement: Figure 4-1 — Shared explained variance of the context-level semantic feature space with each of the other feature spaces for each additional subject. To quantify the amount of overlap between the context-level semantic feature space with each of the four other feature spaces, three models for each pair of feature spaces were fit which included the concatenated feature space and each feature space individually. For each pair of models, the variance explained by each partition in each voxel was projected onto the corresponding cortical flatmaps). Only voxels that were significantly predicted (one-sided permutation test, q(FDR) < 0.05) by each union model are shown.There is substantially lower variance explained by the intersection between the context-level semantic model and the language- and modality-specific feature spaces in the cerebellum than in cortex. The results are largely consistent across subjects. Download Figure 4-1, TIF file. [file ns-JN-RM-0118-21-s09.tif]

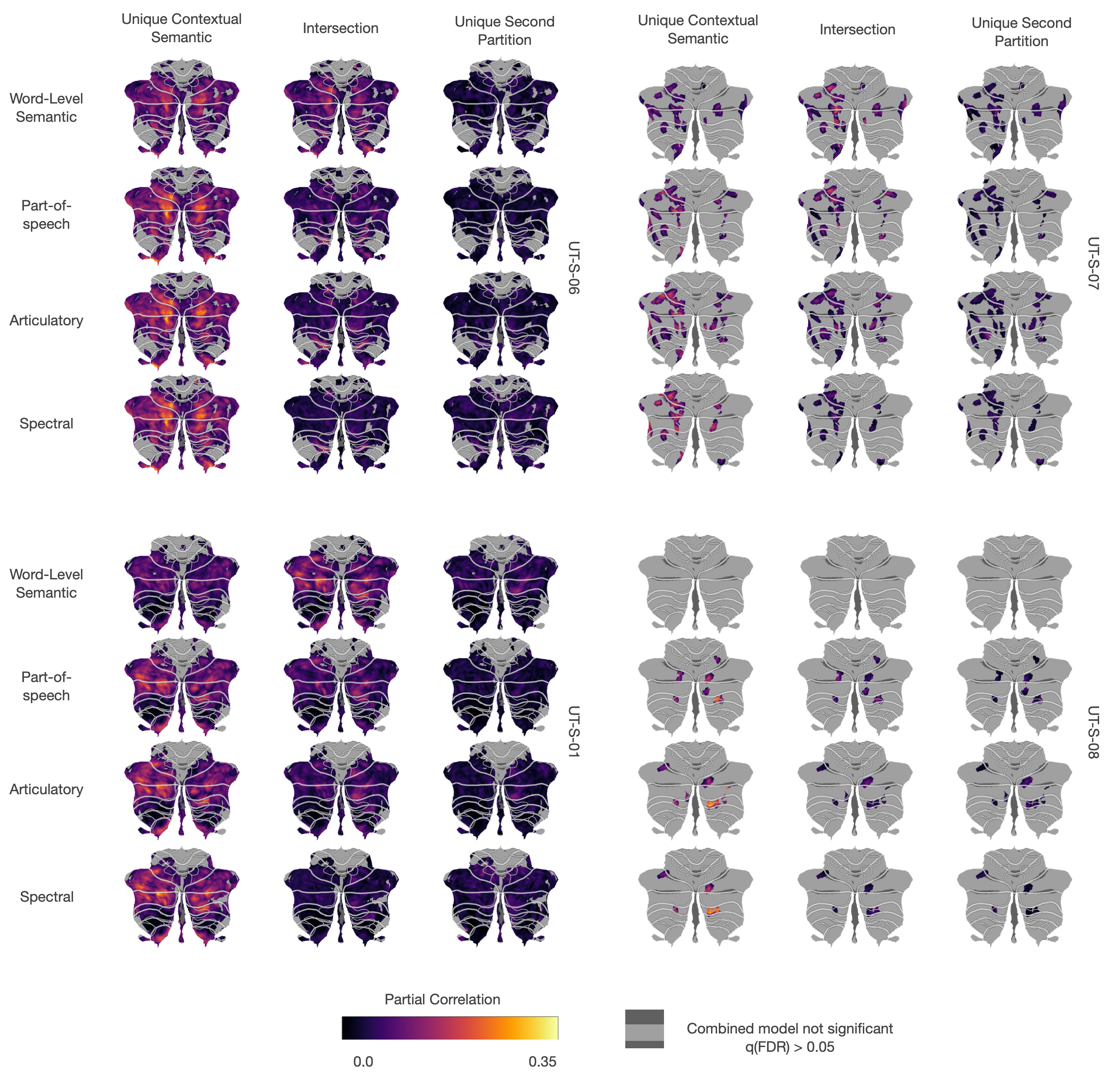

Supplement: Figure 4-2 — Shared explained variance of the context-level semantic feature space with each of the other feature spaces. To quantify the amount of overlap between the context-level semantic feature space with each of the four other feature spaces, three models for each pair of feature spaces were fit which included the concatenated feature space and each feature space individually. For each pair of models, the variance explained by each partition in each voxel was projected onto the corresponding cerebellar flatmaps Only voxels that were significantly predicted (one-sided permutation test, q(FDR) < 0.05) by each union model are shown. There is substantially lower variance explained by the intersection between the context-level semantic model and the language- and modality-specific feature spaces in the cerebellum than in cortex. Additionally, the unique contributions for these models in the cerebellum is approaching zero and is not spatially localized. This lack of spatial localization further supports that there is no hierarchy of language processing in the cerebellum and these results provide strong support for the hypothesis that the cerebellum only represents high level, conceptual features of language, rather than low-level features. This pattern of results appears consistent across subjects Download Figure 4-2, TIF file. [file ns-JN-RM-0118-21-s10.tif]

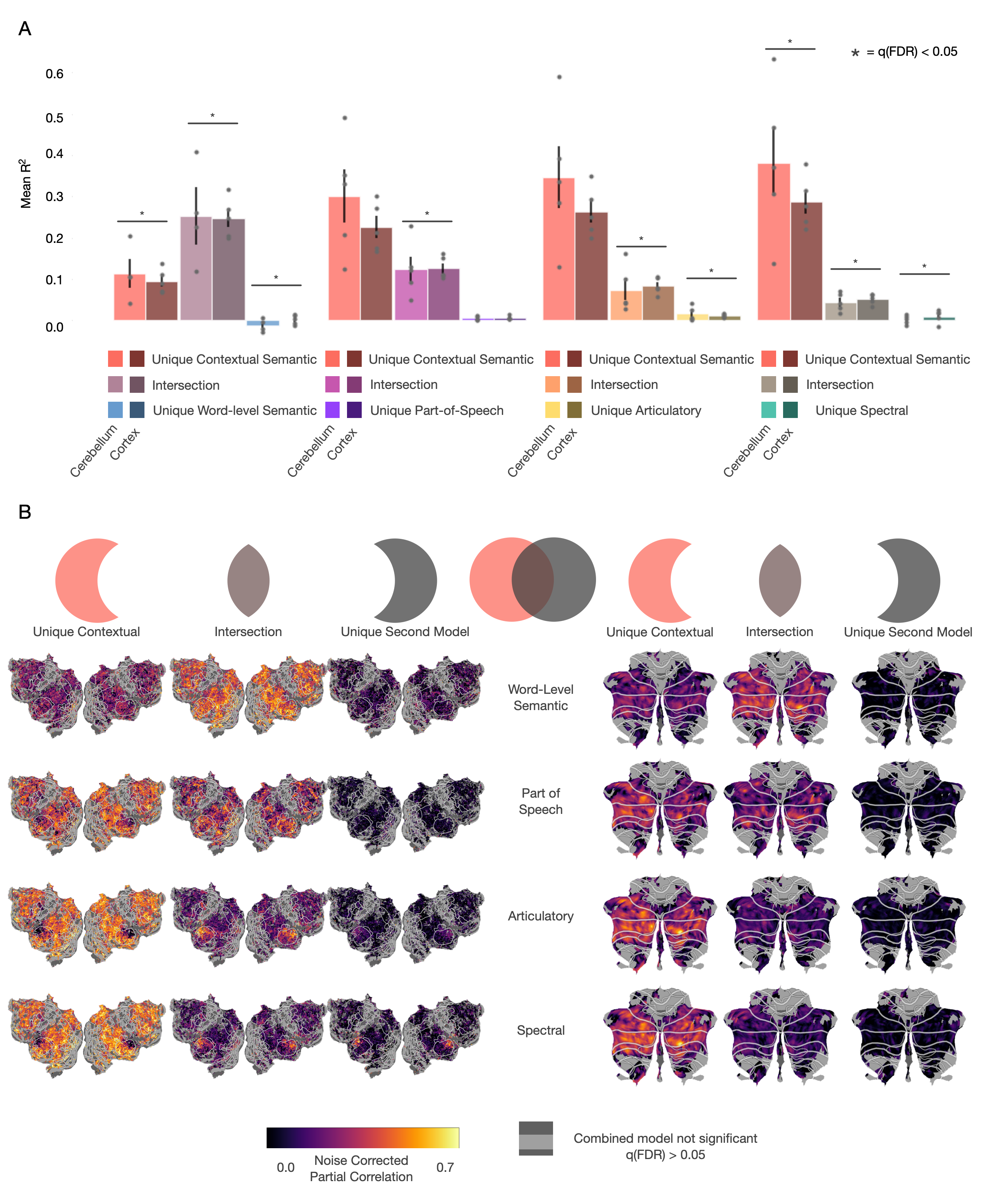

Supplement: Figure 4-3 — Shared explained variance of the context-level semantic feature space with each of the other feature spaces after correcting for signal-to-noise differences. To quantify the amount of overlap between the context-level semantic feature space with each of the four other feature spaces, three models for each pair of feature spaces were fit which included the concatenated feature space and each feature space individually. (A) For each pair of models, the variance uniquely explained by the context-level feature space, uniquely explained by the second feature space, and the intersection between the two for all subjects is compared between the cerebellum and cortex. The unique context-level partition is larger in the cerebellum than in the cortex for all feature spaces (two-sided permutation test, q(FDR) < 0.05). Additionally, the unique partition for each second feature space is significantly smaller in cerebellum than in cortex for every space. This shows that the lower-level feature spaces predict less unique variance in the cerebellum than cortex and further supports the hypothesis that the cerebellum is not representing modality-specific or language-specific information. (B) For each pair of models, the variance explained by each partition in each voxel was projected onto the corresponding cortical and cerebellar flatmaps. These results are noise-ceiling corrected to account for differences in signal-to-noise ratios across the brain. Only voxels that were significantly predicted (one-sided permutation test, q(FDR) < 0.05) by each union model are shown. There is substantially lower variance explained by the intersection between the context-level semantic model and the language- and modality-specific feature spaces in the cerebellum than in cortex. Additionally, the unique contributions for these models in the cerebellum is approaching zero and is not spatially localized. This lack of spatial localization further supports that there is no hierarchy of language processin [file ns-JN-RM-0118-21-s11.tif]

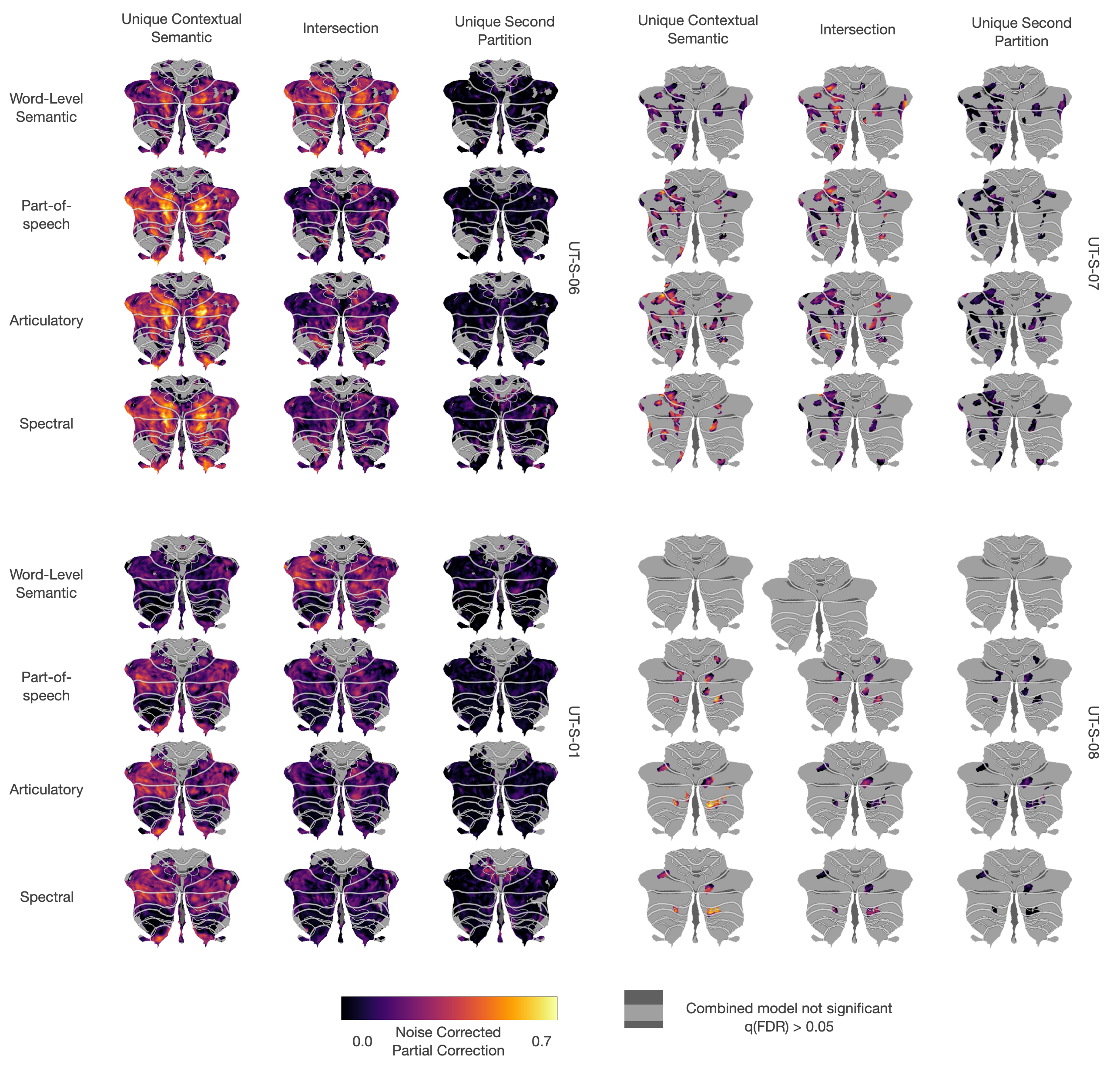

Supplement: Figure 4-4 — Shared explained variance of the context-level semantic feature space with each of the other feature spaces corrected for differences in signal-to-noise in cerebellum. To quantify the amount of overlap between the context-level semantic feature space with each of the four other feature spaces, three models for each pair of feature spaces were fit which included the concatenated feature space and each feature space individually. Correlations were corrected using standard noise-ceiling correction techniques to account for differences in signal-to-noise in cerebellum and cortex. For each pair of models, the variance explained by each partition in each voxel was projected onto the corresponding cerebellar flatmaps. Only voxels that were significantly predicted (one-sided permutation test, q(FDR) < 0.05) by each union model are shown. There is substantially lower variance explained by the intersection between the context-level semantic model and the language- and modality-specific feature spaces in the cerebellum than in cortex. Additionally the unique contributions for these models in the cerebellum is approaching zero and is not spatially localized. This lack of spatial localization further supports that there is no hierarchy of language processing in the cerebellum and these results provide strong support for the hypothesis that the cerebellum only represents high level, conceptual features of language, rather than low-level features. Download Figure 4-4, TIF file. [file ns-JN-RM-0118-21-s12.tif]

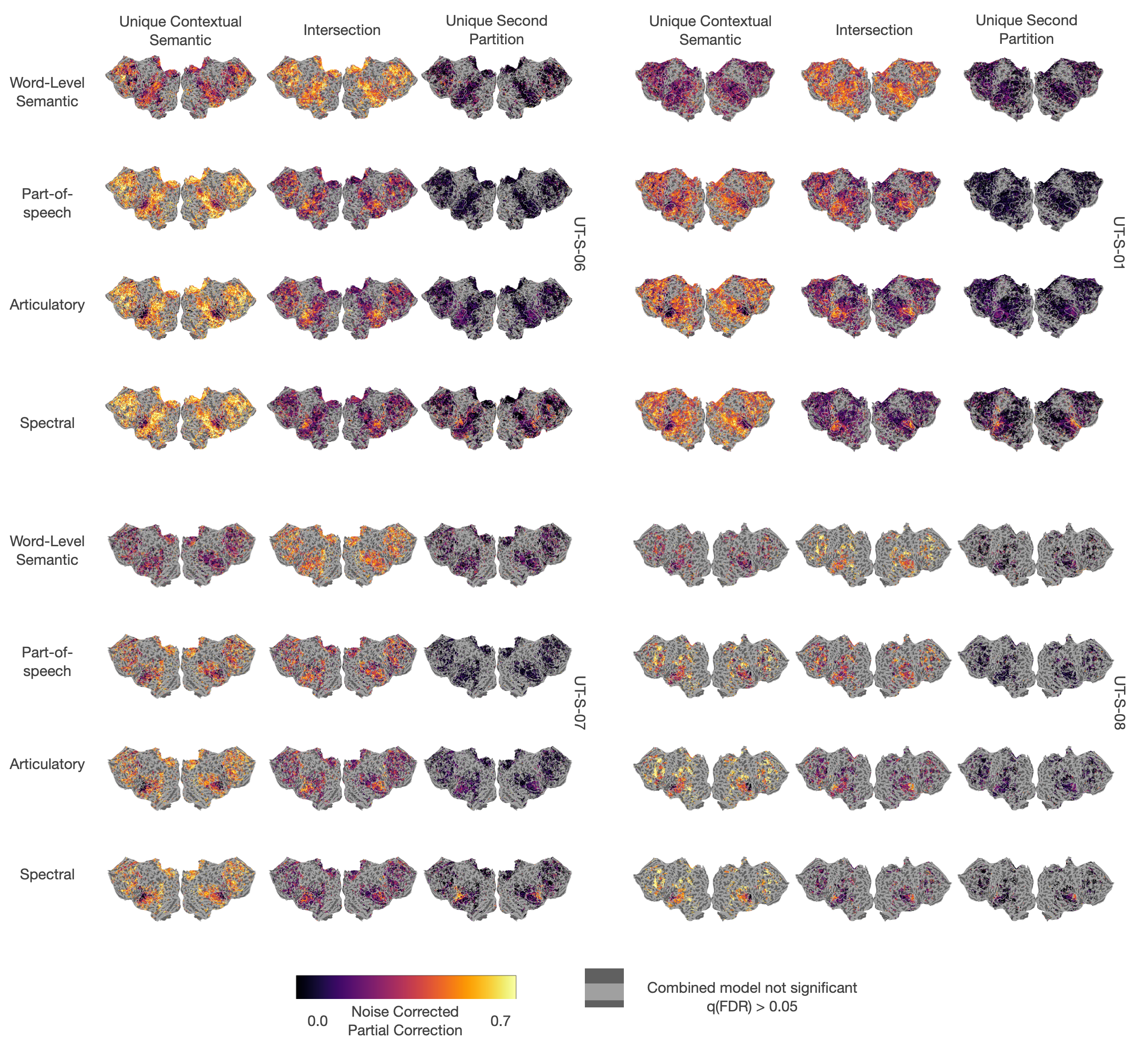

Supplement: Figure 4-5 — Shared explained variance of the context-level semantic feature space with each of the other feature spaces corrected for differences in signal-to-noise in cortex. To quantify the amount of overlap between the context-level semantic feature space with each of the four other feature spaces, three models for each pair of feature spaces were fit which included the concatenated feature space and each feature space individually. For each pair of models, the variance explained by each partition in each voxel was projected onto the corresponding cortical flatmaps. Correlations were corrected using standard noise-ceiling correction techniques to account for variance in the signal-to-noise ratio. Only voxels that were significantly predicted (one-sided permutation test, q(FDR) < 0.05) by each union model are shown. Download Figure 4-5, TIF file. [file ns-JN-RM-0118-21-s13.tif]

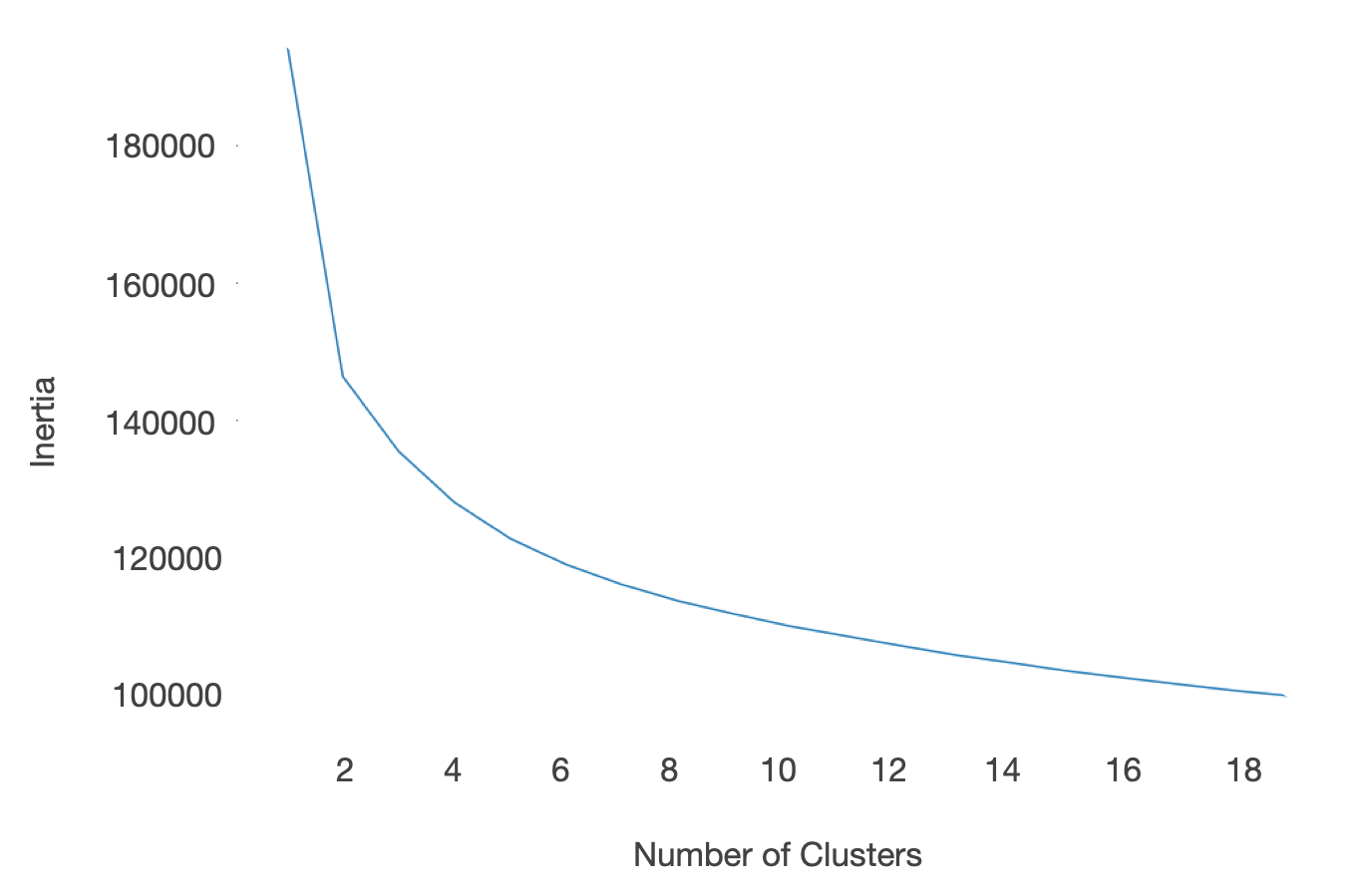

Supplement: Figure 6-1 — Inertia of spherical K-means clustering with increasing number of clusters. To determine the ideal number of clusters to use when clustering the model weights of word-level semantic models, we calculated the inertia at each cluster amount from 1 to 20. There is a clear “elbow point” where the slope of the inertia changes from exponential to linear at 5 clusters, thus that is what we chose to use. However, we did not find any differences in the results using a different number of clusters. Download Figure 6-1, TIF file. [file ns-JN-RM-0118-21-s14.tif]

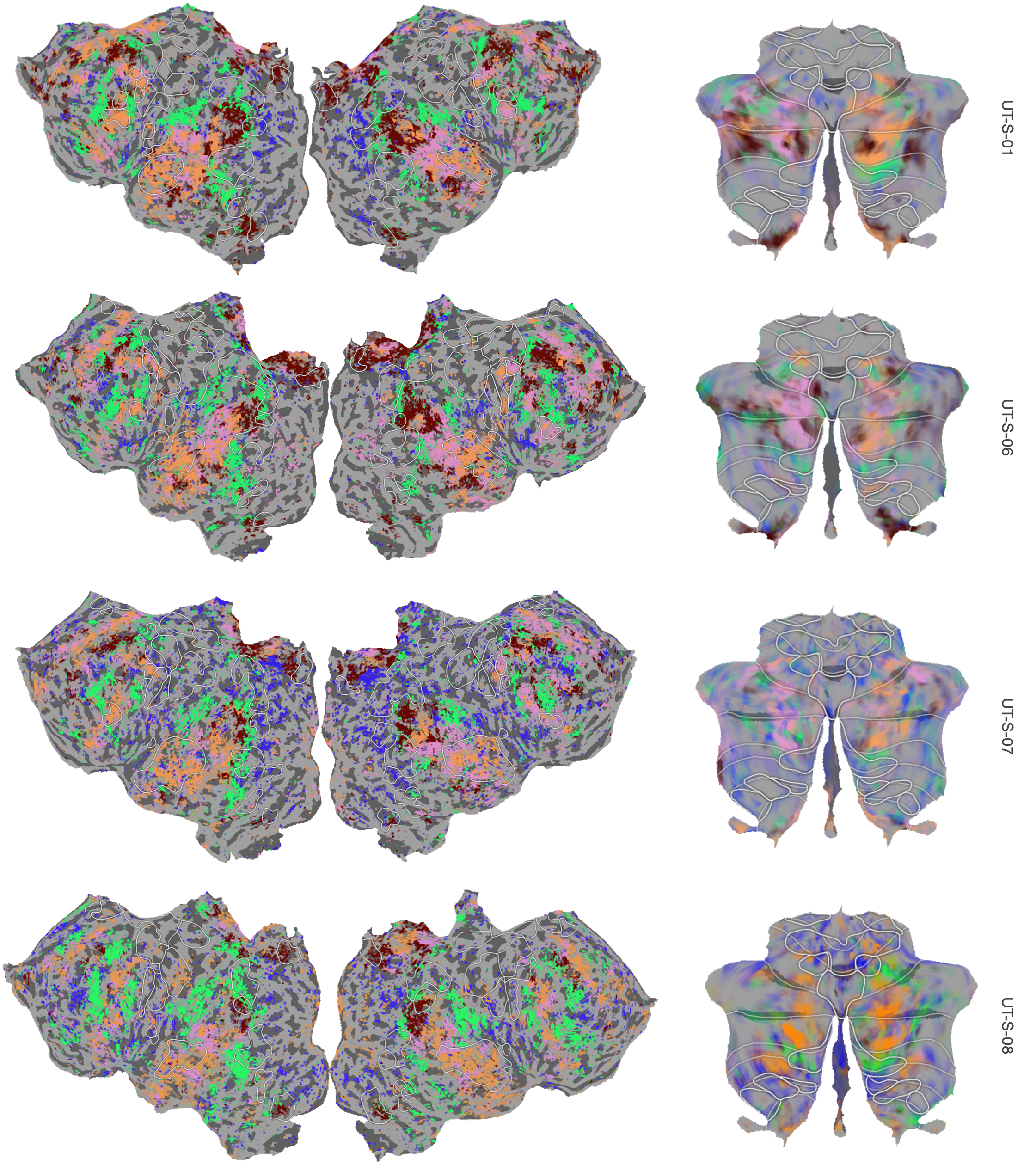

Supplement: Figure 6-2 — Semantic clustering of model weights. To check for differences in semantic representations between the cerebellum and cortex, word-level encoding model weights from both cerebellum and cortex in all subjects were concatenated, including only the top 20% best-predicted voxels. This matrix was then clustered using spherical k-means into 5 clusters, which fell at the inflection point in the inertia graph. For visualization, the centroid for each cluster was transformed into the same RGB space used in Figure 5, and each voxel in that cluster was assigned that color. The cluster distribution for each subject across the cerebellum and cortex are shown. Download Figure 6-2, TIF file. [file ns-JN-RM-0118-21-s15.tif]
